# Non-coding RNAs are key players and promising therapeutic targets in atherosclerosis

**DOI:** 10.3389/fcell.2023.1237941

**Published:** 2023-09-01

**Authors:** Zhun Yu, JinZhu Yin, ZhiTong Tang, Ting Hu, ZhuoEr Wang, Ying Chen, Tianjia Liu, Wei Zhang

**Affiliations:** ^1^ School of Clinical Medical, Changchun University of Chinese Medicine, Jilin, China; ^2^ Cardiology Department, Affiliated Hospital of Changchun University of Chinese Medicine, Jilin, China; ^3^ Department of Massage, Affiliated Hospital of Changchun University of Chinese Medicine, Jilin, China; ^4^ Internal Medicine of Chinese Medicine, Affiliated Hospital of Changchun University of Chinese Medicine, Jilin, China; ^5^ School of Pharmacy, Changchun University of Chinese Medicine, Jilin, China; ^6^ Orthopedics Department, The Third Affiliated Hospital of Changchun University of Chinese Medicine, Jilin, China

**Keywords:** atherosclerosis, ncRNAs, cardiovascular disease, targeted therapy of atherosclerosis, miRNAs

## Abstract

Cardiovascular disease (CVD) is the primary cause of death in humans. Atherosclerosis (AS) is the most common CVD and a major cause of many CVD-related fatalities. AS has numerous risk factors and complex pathogenesis, and while it has long been a research focus, most mechanisms underlying its progression remain unknown. Noncoding RNAs (ncRNAs) represent an important focus in epigenetics studies and are critical biological regulators that form a complex network of gene regulation. Abnormal ncRNA expression disrupts the normal function of tissues or cells, leading to disease development. A large body of evidence suggests that ncRNAs are involved in all stages of atherosclerosis, from initiation to progression, and that some are significantly differentially expressed during AS development, suggesting that they may be powerful markers for screening AS or potential treatment targets. Here, we review the role of ncRNAs in AS development and recent developments in the use of ncRNAs for AS-targeted therapy, providing evidence for ncRNAs as diagnostic markers and therapeutic targets.

## Introduction

Cardiovascular disease (CVD) is the leading cause of death in humans, with over 17 million CVD-related deaths occurring in 2020 (2020; Acosta et al., 2021). CVD mainly includes atherosclerosis (AS), congenital heart disease, arrhythmia, and heart failure, among which AS is the most common (Virani et al., 2021). AS is a chronic inflammatory disease caused by endothelial dysfunction and abnormal lipid metabolism (Ross, 1999; Zhao et al., 2022). In the early stages of AS, intimal low-density lipoprotein (LDL) accumulation triggers inflammation, and macrophages phagocytose the lipids, forming foam cells that trigger the inflammatory response. During this process, the migration and proliferation capacity of many types of cells, such as vascular smooth muscle cells and macrophages, are abnormal (Moore et al., 2013; Wolf and Ley, 2019; Mushenkova et al., 2020). Subsequently, atherosclerotic plaques are formed by inflammatory cells and smooth muscle cell apoptosis, angiogenesis, and thrombogenesis. The prevalence of AS is high and underlies most CVDs, leading to high-mortality-rate manifestations, such as acute coronary syndrome and myocardial infarction (Falk, 2006; Xie et al., 2020). Early AS has no obvious symptoms; thus, early screening for AS is necessary to effectively prevent high-risk CVD (Liu et al., 2018). Imaging remains the primary means for early AS diagnosis, and due to advances in medical technology, some molecular markers shown promise for improving AS screening (Meng H. et al., 2022). Notably, although aging is a major risk factor for AS, with changes in lifestyle habits and dietary structure, there is now a trend toward the development of AS in younger individuals (Libby, 2021; Tyrrell and Goldstein, 2021). Due to the harm of AS to human health, substantial research has been conducted on the mechanisms underlying its occurrence and development to improve the current status of AS diagnosis and treatment. Indeed, epigenetic modifications contribute to AS, such as an abnormal DNA methylation status, altered histone modification levels, and disordered ncRNA expression levels (Xu et al., 2018). NcRNAs are not involved in protein synthesis but can regulate gene expression (Zhang et al., 2016; Bonilauri and Dallagiovanna, 2022; Sandmann et al., 2023).

Long noncoding RNAs (lncRNAs), microRNAs (miRNAs), circular RNAs (circRNAs), ribosomal RNAs, and tRNAs are the most common ncRNAs(Palazzo and Lee, 2015; Zhang Y. et al., 2022; Dragomir et al., 2022). There is increasing evidence that ncRNAs are important in vascular biology, maintaining human health (Jinn et al., 2015; Håkansson et al., 2019; He et al., 2021; Sletten et al., 2021; Farina et al., 2023). Abnormal ncRNA expression can give rise to numerous diseases, including CVD, various cancers, neurodegenerative diseases, and diabetes (Anastasiadou et al., 2018; Mehta et al., 2020; Zhao et al., 2021). NcRNAs have been shown to regulate biological features, biological mechanisms, and phenotypes, such as cell proliferation, apoptosis, and migration capabilities, and have demonstrated potential as therapeutic targets in various diseases (Zhao W. et al., 2018; Ji et al., 2022). In recent years, abnormally expressed ncRNAs have been found to play an important role in various stages of AS progression, including endothelial damage, plaque formation, and plaque instability (Simion et al., 2020; Cui et al., 2022).

Here, we review the abnormal expression of ncRNAs in the occurrence and development of AS and the specific role of key ncRNAs in these processes. We then introduce several ncRNA-targeted treatment methods for AS, exploring the potential of ncRNAs as diagnostic and therapeutic targets in this disease.

### miRNAs participate in AS development and are potential treatment targets

MiRNAs are ncRNAs approximately 20 nucleotides in length. MiRNA is first transcribed into pre-miRNA, which moves from the nucleus to the cytoplasm, undergoes progressive processing, and ultimately matures. Mature miRNAs participate in the formation of RNA silencing complexes (RISCs) and impart RISC targeting by loosely pairing with mRNAs with corresponding miRNA responsive elements (MREs) (Kobayashi and Tomari, 2016; Zealy et al., 2017). Then, miRNAs use RISCs to degrade target mRNA or inhibit its translation, representing a key form of post-transcriptional regulation (Lu and Rothenberg, 2018).

Abnormal miRNA expression has been observed frequently in AS in recent years. Indeed, miRNAs can be used as therapeutic and diagnostic biomarkers and drug targets in AS. The regulatory roles of an increasing number of miRNAs in the occurrence and development of AS are becoming clear (Table 1).

**TABLE 1:** The role of miRNAs in AS.

miRNA	The expression level of miRNA in AS	Function in AS	References
miR-342-5p	Up	Promotes endothelial cell apoptosis	Xing et al. (2020)
miR-19b	Up	Promotes inflammatory response	Wang et al. (2021)
miR-202-5p	Up	Promotes apoptosis of macrophages	Xu et al. (2023)
miR-217	Up	Increase blood pressure and exacerbate atherosclerosis	de Yébenes et al. (2020)
miR-124-3p	Up	Inhibit proliferation and promots apoptosis of macrophage	Zhai et al. (2020)
miR-144	Up	Influence intimal hyperplasia	Chen et al. (2018), Lian et al. (2022)
miR-126-5p	Down	Attenuates endothelial cells apoptosis and promotes endothelial cells proliferation	Schober et al. (2014), Santovito et al. (2020a)
miR-133a	Down	Inhibits proliferation and promotes apoptosis of VSMCs and promotes differentiation of VSMCs	Cipollone et al. (2011), Gao et al. (2014), Shi et al. (2019)
miR-143/145	Down	Inhibits VMSCs migration and promotes VMSCs differentiation	Li et al. (2018), Ni et al. (2021), Vacante et al. (2021)
miR-155	UP	It inhibits macrophage proliferation in early AS and efferocytosis in advanced AS	Wei et al. (2015)
miR-21	Up	Regulates circadian apoptosis of macrophages and promotes migration and proliferation of VSMCs	Raitoharju et al. (2011), Zhu et al. (2019a), Schober et al. (2021)
miR-330-3p	Down	Reduces endothelial cell apoptosis, promotes endothelial cell proliferation and migration	Shan et al. (2023)
miR-1914-5p	Down	Inhibits monocyte adhesion and migration	Toriuchi et al. (2023)
miR-147a	Down	Weakens monocyte adhesion and increases the stability of AS plaques	Chen et al. (2023)
miR-205-5p	Down	Promotes apoptosis and inhibits migration of vascular smooth muscle cells	Huang et al. (2023)
miR-455-5p	Down	Reduces endothelial cell scorching and vascular inflammation	Li et al. (2023)
MiR-148a-3p	Down	Reduces macrophage apoptosis and inflammation	Wang et al. (2022a)
miR-186-5p	Down	Reduces lipid accumulation in macrophages	Ding et al. (2022)

Differentially expressed miRNAs in AS progression can now be more efficiently identified from tissue or blood samples due to advances in sequencing technologies, enabling the analysis of potential key miRNAs in AS and their downstream mechanisms (Moreau et al., 2021; Dragomir et al., 2022; Santovito and Weber, 2022). Albuminuria is a marker of endothelial dysfunction. Screening of miRNAs in the plasma of hypertensive patients with albuminuria symptoms revealed that miR-126-3p was abnormally elevated and associated with cardiovascular events; thus, this miRNA is a potential molecular marker for AS (Martinez-Arroyo et al., 2023). miR-126-5p is also involved in endothelial cell protection via autophagy in AS (Santovito et al., 2020a). In a recent study of AS in 16 baboons, blood and common iliac artery (CIA) samples were collected before and after 2 years of high cholesterol and high-fat diets, and miRNA expression levels were analyzed to identify potential molecular markers of human AS. miR-17-5p and miR-146a-5p, which are upregulated in both fatty streak and fibrous plaque lesion types, were revealed as possible key players in AS development (Karere et al., 2023). Another previous report indicated that miR-17-5p and miR-146a-5p demonstrate potential in the clinical diagnosis of AS (Sharma et al., 2022). miR-342-5p is significantly overexpressed in damaged endothelial cells and significantly affects apoptosis levels in oxidatively damaged endothelial cells by regulating PPP1R12B (Xing et al., 2020). miR-19b expression was found to be elevated in the endothelial cells and arterial tissues of an ox-LDL-induced AS mouse model and promoted inflammation, thus participating in AS development by inhibiting the ubiquitination of NF-κB/p65 by PPARγ(Wang et al., 2021). Abnormally elevated levels of miR-202-5p have been found in AS tissues and to induce macrophage apoptosis by inhibiting Bcl-2 expression levels, thereby promoting AS plaque formation and reducing fibrous cap thickness *in vivo*, which in turn increased the risk of plaque rupture (Xu et al., 2023). In addition, reduced expression of some miRNAs has been observed during the AS process, suggesting that these miRNAs may have a potential inhibitory effect on AS. Reduced miR-330-3p expression levels and increased AQP9 expression levels have been observed in an AS mouse model. Further experimental results indicated that miR-330-3p could reduce endothelial cell apoptosis and promote endothelial cell proliferation and migration by inhibiting AQP9 (Shan et al., 2023). The recruitment of inflammatory cells is an important event in AS plaque formation. Monocytes adhere to the vessel wall and gradually migrate to the subendothelial layer in the inflammatory state, during which IL-1β upregulates the expression level of cell adhesion molecules; this effect is achieved by inhibiting miR-1914-5p (Toriuchi et al., 2023). When miR-1914-5p was overexpressed, the migration ability of monocytes induced by IL-1β across the endothelial cell layer was significantly inhibited. Ox-LDL-induced AS resulted in a significant downregulation of miR-147a expression levels during AS, which led to elevated ZEB2 levels that mediated monocyte adhesion to endothelial cells, exacerbating lipid accumulation and AS plaque formation; in contrast, elevated miR-147a expression could stabilize AS (Chen et al., 2023).

Numerous studies have focused on new drugs for AS and their key underlying mechanisms, revealing the importance of miRNA expression regulation in the associated pathways. Sodium butyrate, a product of intestinal flora, has a therapeutic effect on AS. A previous report revealed that sodium butyrate in an AS model increased the expression levels of 29 miRNAs, including miR-7a-5p, and reduced the expression of 24 (Ma et al., 2023). This finding suggests that sodium butyrate can regulate miRNA expression in AS. Icariside (ICA), the main active ingredient of Epimedium, can produce significant therapeutic effects in animal models of AS, reducing lipid accumulation and plaque formation in blood vessels. Further studies demonstrated that ICA elevated miR-205-5p expression (Zhu and Ren, 2022), promoted endothelial cell apoptosis, and inhibited their migration; silencing miR-205-5p reversed this inhibitory effect, demonstrating miR-205-5p upregulation is a potential approach for AS treatment (Huang et al., 2023). There is evidence that miR-217 can inhibit apoptosis through the TLR4/PI3K/Akt/NF-κB pathway in atherosclerotic endothelial cells in a rat model (Zhang et al., 2020). Moreover, miR-124-3p overexpression inhibits macrophage proliferation and apoptosis by downregulating MEKK3 expression in a mouse model of AS (Zhai et al., 2020). In addition, numerous studies have indicated that miRNAs are strongly expressed cell lines associated with AS; for example, miR-126-5p in human endothelial cells promotes cell proliferation and reduces apoptosis by inhibiting caspase-3 and Dlk1 (Schober et al., 2014; Santovito et al., 2020a); miR-133a and miR-145 in human VSMCs inhibit cell migration and promote contractile phenotype (Cipollone et al., 2011; Gao et al., 2014; Li et al., 2018; Shi et al., 2019); miR-21 and miR-155 are present in human and mouse monocytes/macrophages, and the former regulates circadian apoptosis in macrophages, whereas the latter exerts inhibitory in early AS but promotional effects in advanced AS (Raitoharju et al., 2011; Wei et al., 2015; Schober et al., 2021). miR-144 knockdown has been shown to attenuate intimal hyperplasia in VSMCs(Lian et al., 2022). The above findings indicate that miRNAs play a role in AS.

MiRNAs, key post-transcriptional regulation factors, change during AS development, likely affecting downstream gene expression homeostasis and AS. Thus, drugs could be developed to target miRNAs that regulate gene expression in AS. In conclusion, abnormally expressed miRNAs are potential diagnostic biomarkers and potential therapeutic targets in AS, and reversing abnormal miRNA expression levels may be a powerful AS prevention or treatment modality.

### lncRNAs act as molecular sponges in the AS process

Single-stranded RNAs greater than 200 nucleotides in length are classified as lncRNAs. Some lncRNAs are processed similarly to mRNAs, transcribed by RNA pol III, or capped/polyadenylated (Simion et al., 2019). The competing endogenous RNA (ceRNA) hypothesis posits that lncRNAs can competitively bind to miRNA and regulate the expression of miRNA target genes (Salmena et al., 2011). Research on lncRNAs continues to advance, and our knowledge of their functions has been enriched, suggesting that many diseases are closely related to abnormal lncRNA expression (Table 2).

**TABLE 2:** The role of lncRNAs in AS.

lncRNAs	The expression level of lncRNAs in AS	Function in AS	References
MAARS	Up	Promotes apoptosis of macrophages	Simion et al. (2020)
RASSF8-AS1	Up	Promotes vascular smooth muscle cell proliferation and inhibits apoptosis	Song et al. (2023)
CARMN	Down	Regulates vascular smooth muscle cell proliferation, migration and differentiation; increases the area and volume of strong AS plaques	Dong et al. (2021), Ni et al. (2021), Vacante et al. (2021)
AI662270	Up	Accelerates foam cell formation	Hong et al. (2023)
NIPA1-SO	Down	Inhibits monocyte adhesion and foam cell formation	Jiang et al. (2023)
Lnc_000048	UP	Accelerates inflammation and collagen degradation and reduces plaque stability	Zhang et al. (2023a)
lncR-GAS5	UP	Inhibits autophagy in endothelial cells	Fan et al. (2023)
Punisher	Down	Inhibits vascular smooth muscle cell apoptosis by regulating mitochondrial homeostasis	Yang et al. (2022)
MDRL	Down	Inhibits NLRP3 inflammasome activation and apoptosis of vascular smooth muscle cells	You et al. (2022)
Gaplinc	UP	Causes vascular endothelial cell scorching	Tang et al. (2022)
APPAT	Down	Inhibits the proliferation and migration of vascular smooth muscle cells	Meng et al. (2022a)

The lncRNA CARMN, upstream of miR-143 and miR-145, has been found to be downregulated in late AS plaques. CARMN knockdown downregulated miR-143 and miR-145 expression and accelerated AS progression in mice, increasing the area and volume of AS plaques and producing a late AS phenotype (Dong et al., 2021; Ni et al., 2021; Vacante et al., 2021). Moreover, CARMN has been considered a regulator of mouse and human VSMC plasticity in AS (Dong et al., 2021; Ni et al., 2021). In these studies, CARMN promoted the contractile phenotype of VSMCs through miR-143/145, whereas regulation of VSMCs proliferation was independent.

NcRNAs are known to be involved in cellular biology processes in AS, such as cell apoptosis (Kockx and Herman, 2000). MALAT1 can mediate cell autophagy and affects plaque inflammation in AS (Zhu Y. et al., 2019; Cremer et al., 2019). LncRNA RASSF8-AS1 is highly expressed in the serum of AS patients and can promote ATG7-mediated autophagy through the competitive combination of miR-188-3p, promote the proliferation ability and anti-apoptotic activity of smooth muscle cells, and play an important role in the development of AS plaques (Song et al., 2023). A recent study identified the macrophage-specific lncRNA MAARS and observed a 270-fold increase in its expression level in the aortic intima during AS progression and a 60% decrease during AS regression, indicating that this lncRNA has high clinical value as a biomarker for AS diagnosis (Simion et al., 2020). In the aforementioned study, knocking out MAARS resulted in a 52% reduction in AS lesions in LDLR^−/−^ mice; MAARS can interact with the RNA binding protein HuR and thus regulate macrophage apoptosis levels. Knocking down MAARS increases the exocytosis of macrophages and reduces their apoptosis level, thus reducing AS plaque necrosis.

In addition, the lncRNA AI662270 has been observed to be specifically enriched in macrophages during AS. AI662270 overexpression promotes lipid accumulation, reduces the cholesterol outflow of macrophages, and accelerates the formation of foam cells, yielding an AS-promoting effect. AI662270 knockdown exerts a therapeutic effect on AS (Hong et al., 2023). Moreover, there is evidence that MIAT can affect lesion formation and plaque destabilization in AS. MIAT regulates smooth muscle cell proliferation and apoptosis as well as contributes to the transformation of smooth muscle cells into inflammatory macrophage-like cells (Fasolo et al., 2021).

Due to the broad regulatory role of lncRNAs, their abnormal expression often affects the expression levels of multiple downstream genes. LncRNAs may be upstream of other factors in the complex mechanism of AS development and have greater potential as a diagnostic marker for this disease. In addition, a large body of evidence suggests that regulating lncRNA expression can have therapeutic effects on AS and that targeting lncRNAs in AS treatment may produce therapeutic effects through multiple downstream pathways. Further exploration of these specific mechanisms will enrich the theoretical basis of AS.

### circRNAs play a functional role in AS

**FIGURE 1:**
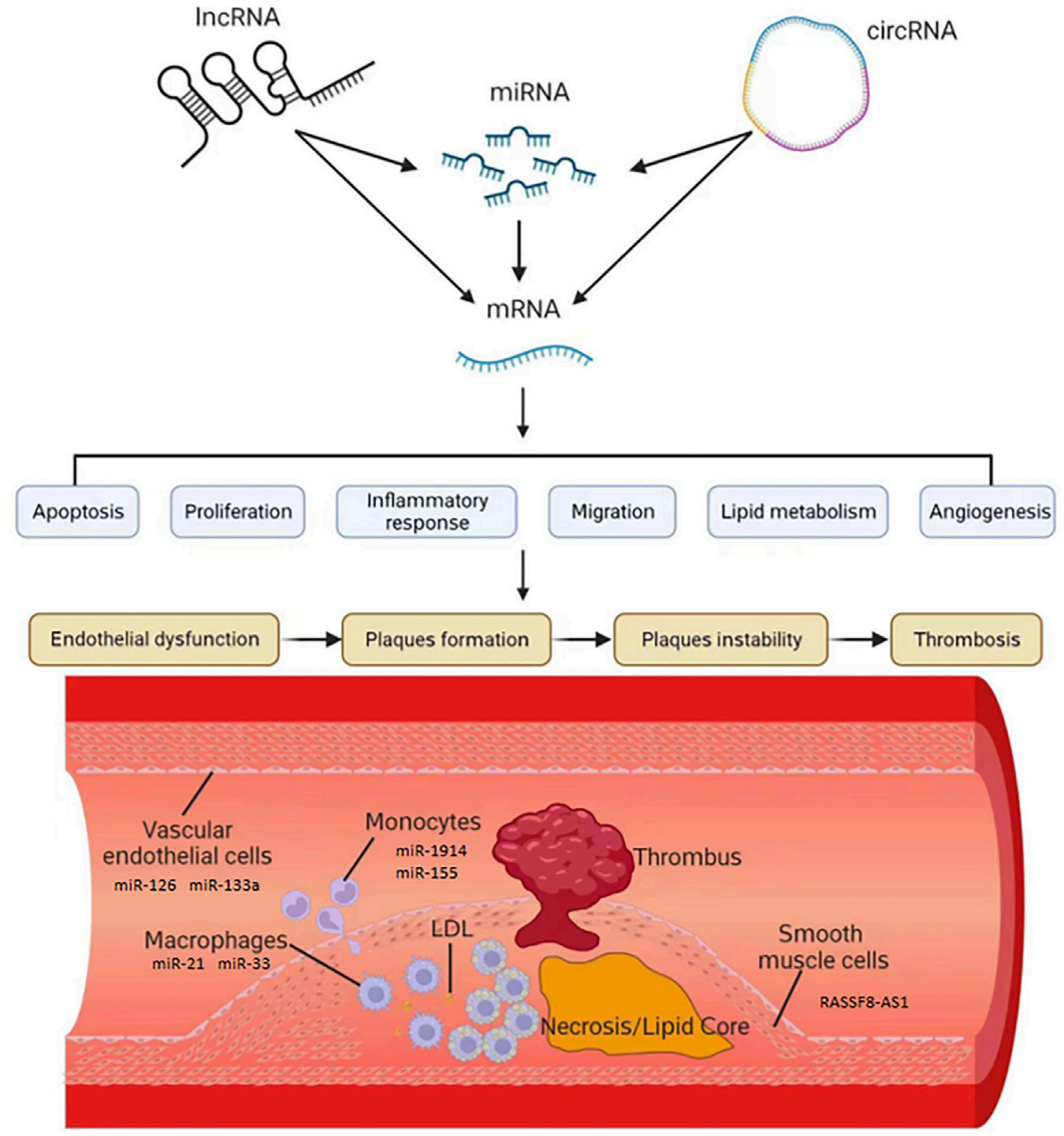
The role of ncRNAs in the development of AS. The expression of mRNA which was related to AS was regulated by lncRNA, miRNA and circRNA. These ncRNAs, such as miR-126, miR-133a, miR-21, miR-33, miR-1914,miR-155 and lncRNA RASSF8-AS1, could influence apoptosis, proliferation, inflammatory, migration, lipid metabolism and angiogenesis in vascular endothelial cells, macrophages, monocytes and smooth muscle cells.

Unlike other ncRNAs, circRNAs exhibit a loop structure. The closed-loop structure of circRNAs provides stability in the presence of RNA nucleic acid exonucleases (Zhao K. et al., 2018). Similar to lncRNAs, circRNAs regulate target mRNA expression levels and influence protein levels by competitively binding to miRNAs(Abdelmohsen et al., 2017). As research has progressed, understanding of circRNAs has gradually increased; however, most circRNAs’ functions remain unclear (Li et al., 2019). Multiple circRNAs have been shown to be differentially expressed during AS progression and, similar to miRNAs and lncRNAs, are involved in various stages of AS occurrence and development (Figure 1). The expression of circRNAs demonstrates temporal and spatial specificity, which, combined with their stable expression characteristics, affords them good potential as molecular markers of AS (Singh et al., 2022). In addition, circRNAs play an important role in gene regulatory networks, and reversing the abnormal expression of key circRNAs in AS may be a potential therapeutic option. Therefore, exploring the key circRNAs in AS and analyzing their role is important for developing diagnostic and treatment approaches for this disease (Table 3).

**TABLE 3:** The role of circRNAs in AS.

circRNA	The expression level of circRNA in AS	Function in AS	References
circ_0090231	Up	Promotes endothelial cell scorching	Ge et al. (2021)
circ_0026218	Down	Reduces endothelial cell apoptosis and inhibits inflammatory response	Yang et al. (2023a)
circRNA_06206	Down	Inhibits angiogenesis	Yuan et al. (2023)
circ_0008896	Up	Promotes endothelial cell apoptosis, angiogenesis and inflammatory response	Liu et al. (2023)
circ_0000280	Down	Inhibits proliferation of smooth muscle cells	Wang et al. (2022b)
circ COL1A1	Up	Promotes the conversion of vascular smooth muscle cells from a contractile to a synthetic phenotype	Ye et al. (2023)
circ_0007478	Up	Promotes foam cell formation	Ye et al. (2022)
circ_0007478	Up	Inhibits endothelial cell viability and promotes endothelial damage	Zhang et al. (2022a)
circNMD 3	Down	Reduces endothelial cell inflammation and oxidative stress	Xiu et al. (2022)
circ_0030042	Up	Promotes the proliferation and migration of vascular smooth muscle cells	Ma et al. (2022)

The potential role of certain circRNAs in AS has been recognized, and sequencing has been used to directly screen differentially expressed circRNAs to explore AS diagnostic markers. Abnormally elevated circRNA has_circ_0126672 was found in the gene expression profile associated with coronary artery disease. This circRNA can competitively adsorb numerous miRNAs, including miR-145-5p, and is closely associated with AS (Rafiq et al., 2023). Feng Zhang et al. prepared AS rabbit models using a high-fat diet, screened the differentially expressed circRNAs, miRNAs, and mRNAs by RNA-seq and mapped the ceRNA network, concluding that seven circRNAs, including ocu-cirR-novel-18038, were associated with AS by gene function enrichment analysis (Zhang et al., 2018). In addition, circZNF292 in endothelial cells and circLRP6 in VSMCs have been demonstrated to influence endothelial cell shape and vascular diseases in both humans and mice (Hall et al., 2019; Heumüller et al., 2022). CircZNF292 regulates endothelial cell flow responses, while circLRP6 participates in AS by acting as a molecular sponge of miR-145.

After screening for differentially expressed circRNAs in the AS process, further analysis of the role of these circRNAs in AS facilitates the development of therapeutics. In an AS cell model prepared using ox-LDL treatment, circ_0090231 was found to promote the progression of AS by competitively adsorbing miR-635, increasing NLRP3 expression levels and triggering higher levels of cell damage and cell scorching (Ge et al., 2021). In addition, ox-LDL treatment significantly reduced the expression level of circ_0026218, and overexpression of this circRNA increased the expression level of SIRT6 by adsorbing miR-338-3P, enhancing endothelial cell viability and inhibiting the inflammatory response and apoptosis. In contrast, miR-338-3p overexpression reversed the protective effect of circ_0026218 on endothelial cells. Thus, the circ_0026218/miR-338-3p/SIRT6 axis is closely associated with endothelial injury in the early stages of AS (Yang L. et al., 2023).

Angiogenesis is a key event in AS progression and increases AS plaque instability, further elevating AS-related CVD risk. A recent study identified a novel circRNA_06206, circSCRG1, and found that its expression level decreased after ox-LDL treatment and increased after treatment with drugs blocking endothelial cell angiogenesis. Further studies revealed that circSCRG1 has a role in stabilizing AS plaques by competitively adsorbing miR-1268b to regulate NR4A1 expression levels and thereby inhibit angiogenesis (Yuan et al., 2023).

Some circRNAs are expressed at higher levels during the AS process, and further mechanistic exploration has revealed that they could play a role in promoting AS; in contrast, certain circRNAs show decreased expression levels during AS and perform several functions, including the protection of endothelial cells and inhibition of apoptosis. In addition, the stability of circRNAs increases their potential for use as biomarkers versus other ncRNAs. In conclusion, as research on circRNAs in AS continues to unfold, circRNAs as diagnostic markers and therapeutic targets may help alleviate the burden associated with AS.

### AS-targeted therapeutic approaches using ncRNAs as targets

**FIGURE 2:**
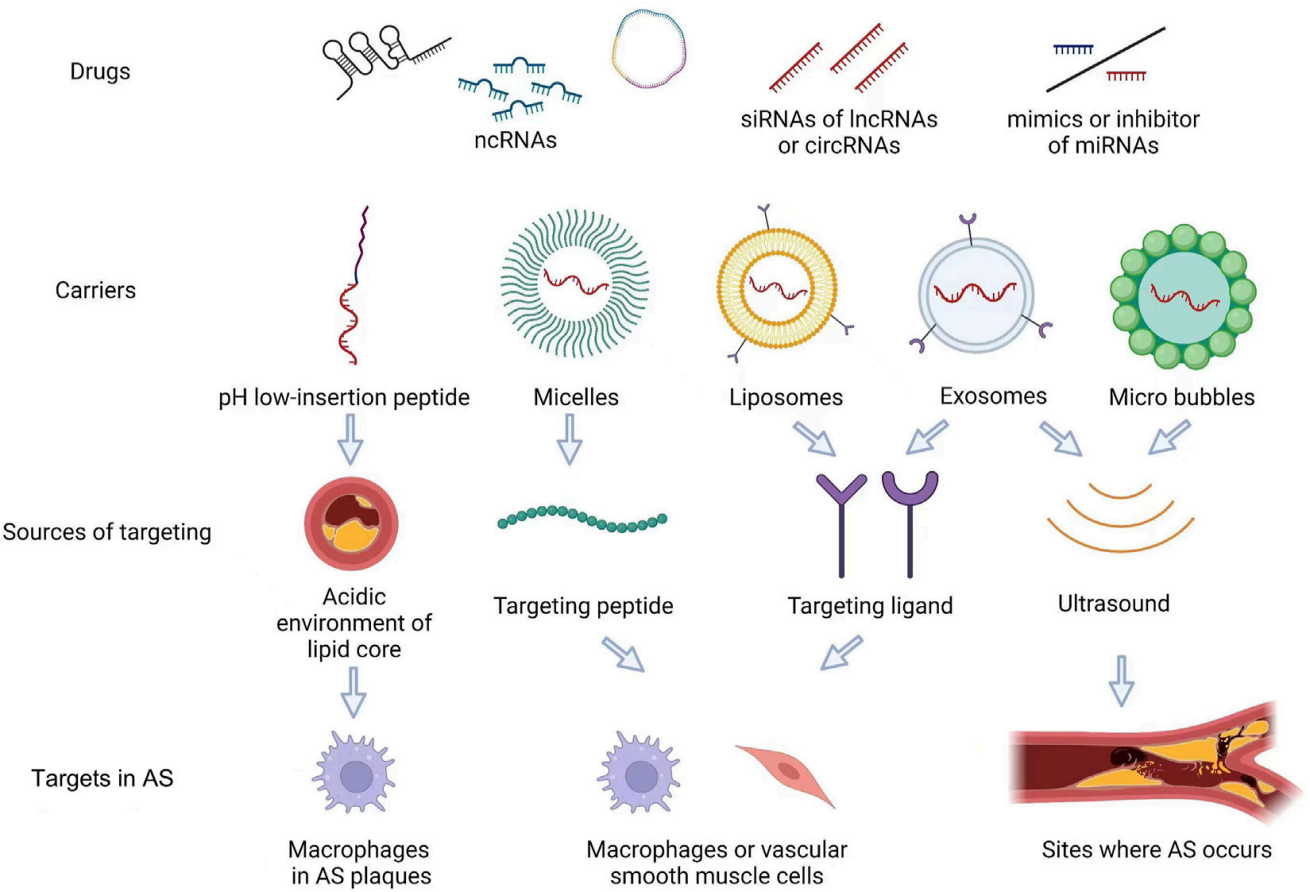
AS-targeted therapeutic approaches using ncRNAs as targets. As potential drugs, a few of siRNA of lncRNAs or circRNAs, mimics or inhibitor of miRNAs, can be used in AS. Furtherly, the carriers which were micelles, liposomes, exosomes and micro bubbles, can deliver ncRNAs for AS therapy. These carries can target lipid core, peptide, ligand in Macrophages and VSMCs. Exosome and micro bubbles can used in the sites where the AS occurs by ultrasound.

With the development of medical technology, precision and personalized medicine approaches are increasingly becoming the main development trend in clinical medicine (Wang et al., 2018). Some drugs have been demonstrated to regulate ncRNA expression related to AS. Furthermore, as potential drugs, lncRNAs and miRNAs might play a role in AS treatment (Saenz-Pipaon and Dichek, 2022). Regarding ncRNAs in precision and personalized medicine, carriers should have the characteristics of low cytotoxicity, non-immunogenicity, and ease of mass production (Nam et al., 2015). Thus, peptides, micelles, liposomes, exosomes, and microbubbles have been assessed as carriers to deliver ncRNAs for AS therapy (Terashima et al., 2018; Aoki et al., 2020; Scholz et al., 2022) (Figure 2).

Exosomes are cell-derived vesicles, typically 50–200 nm, containing rich RNA, DNA, protein, and lipid content. Exosome content secreted by different types of cells varies in composition (Shao et al., 2018). Exosomes play a dual role in AS, on the one hand worsening AS through the contents they carry. For example, exosomes secreted by nicotine-stimulated macrophages contain large amounts of miR-21-3p and increase the migration and proliferation ability of vascular smooth muscle cells through miR-21-3p, thus accelerating the progression of AS (Zhu J. et al., 2019). On the other hand, a fraction of exosomes can have therapeutic effects on AS. For example, adipose-derived MSC exosomes can inhibit miR-342-5p expression and may thereby reduce endothelial cell damage in AS (Xing et al., 2020). Since exosomes themselves may contain therapeutic ncRNAs, nucleotide drugs, such as siRNAs, can be further artificially piggybacked by electroporation to enhance their therapeutic effects (Alvarez-Erviti et al., 2011; Familtseva et al., 2019). With the development of molecular engineering technology, exosomes with higher targeting capabilities can be generated by adding ligands for the targeted treatment of AS (Liang et al., 2021). For example, a recent trial evaluated prepared engineered M2 macrophage-derived exosomes with higher inflammatory tropism and anti-inflammatory effects that could better carry the contents for the treatment of AS (Wu et al., 2020).

Liposomes are lipid bilayer particles with good biocompatibility and bioavailability and allow for modifications on their surface to increase stability and targeting within the plasma (Yan et al., 2019; Scheideler et al., 2020). Liposomes are now widely used to deliver ncRNA drugs; in a recent study miR-146a encapsulated into liposomes demonstrated increased stability that could be preserved for over 2 months, reducing inflammation and decreasing foam cell production (Ho et al., 2023).

Several other types of nanocarriers can be used to deliver ncRNAs for targeted therapy in AS. A novel water-soluble membrane molecule pH low-insertion peptide (pHLIP) was recently proposed as a carrier for AS-targeted therapy based on the acidic environment of the AS lipid core. pHLIP was used to carry antisense oligonucleotides of miR-33-5p to target macrophages in AS plaques without the side effects of systemically reducing miR-33-5p. This study demonstrated that pHLIP could be an excellent vector for miRNA-targeted therapy *in vivo* and successfully reduced lipid accumulation in macrophages by inhibiting miR-33-5p expression, promoting AS regression and increasing AS plaque stability (Zhang X. et al., 2022).

In contrast to the pH-responsive carriers mentioned above, carriers actively targeting AS also exist. In one study, monocyte chemotactic protein-1 (MCP-1) peptide was used to synthesize peptide amphiphile micelle (PAM), which can target monocytes in AS, and MCP-1/C-C motif chemokine ligand 2 (CCL2) was added to it to target miR-145 to vascular smooth muscle cells through its interaction with C-C chemokine receptor-2 (CCR2), which is enriched in vascular smooth muscle cells (Chin et al., 2021). In the aforementioned study, intravenous miR-145 micelles successfully inhibited nearly half of the lesion growth in an early AS model and inhibited AS plaque growth 35% more than free miR-145 in a mid-stage AS model. In a follow-up study, miR-145 micelles were shown to be effective in the long-term treatment of AS *in vivo* (Chin et al., 2023).

Ultrasound-targeted microbubble destruction (UTMD) is another a promising technique for targeted drug delivery that uses ultrasound to induce the rupture of drug-carrying microbubbles at the target site, and this approach allows for perforation of the cell membrane to increase the efficiency of drug delivery (Zhou et al., 2022). Yu Wu et al. synthesized cationic microbubbles and encapsulated miR-145 within them to target its release in vascular smooth muscle cells using ultrasound. The *in vitro* findings demonstrated that this approach significantly increased the transfection efficiency of miR-145; *in vivo*, this approach reduced AS plaque size by nearly half that achieved by direct treatment with free miR-145 (Wu et al., 2023). In addition, UTMD technology can be applied to exosomal vectors to achieve targeted release of the mounted ncRNA (Sun et al., 2020).

Medical developments have revealed that non-targeted therapeutic drugs sometimes result in side effects that cannot be ignored. Especially in gene therapy, the overall regulation of certain genes may lead to more serious consequences for patients. As a result, there has been a concerted effort to develop targeted therapies for a wide range of diseases, including gene therapy. As the number of specific molecules in key cells in AS grows, theoretically, non-viral vectors for AS targeting could also be gradually improved, allowing the more precise delivery of nucleotide drugs to regulate ncRNA expression levels in AS treatment.

## Conclusion

AS is the most common disease of the cardiovascular system, and its ability to induce a variety of fatal CVDs makes AS a constant threat to patients’ life and health. Current AS treatments are pharmacological or surgical, and improving lifestyle habits can help patients with less severe AS. Statins are commonly used to treat AS and have cholesterol-lowering, anti-thrombotic, and anti-inflammatory effects (Min et al., 2013; Vadali and Post, 2014). Antiplatelet agents, hypotensive drugs, and hypoglycemic drugs are also often used in the treatment of AS, and some plant extracts have also demonstrated therapeutic effects; however, despite the continuous development of AS therapies, none are currently curative (Ruan et al., 2022; Shen et al., 2022). Thus, it is important to continue exploring the mechanisms underlying AS development. Currently known risk factors for AS include age, unhealthy diet, smoking, and lack of exercise (Lechner et al., 2020; Tyrrell and Goldstein, 2021). The aging-related miR-217 has been found to be overexpressed in the plasma of patients with CVD. A recent study specifically knocked in miR-217 into endothelial cells in an AS-promoting mouse model, demonstrating the role of miR-217 in reducing NO and promoting endothelial dysfunction, thus exacerbating the AS process, explaining the cause of aging-induced AS to some extent (de Yébenes et al., 2020). miR-124-3p expression levels were significantly higher in smokers than in nonsmokers and past smokers, and increased miR-124-3p expression levels were associated with AS due to the altered monocyte phenotype caused by miR-124-3p overexpression (de Ronde et al., 2017). Thus, more detailed screening of smokers for AS risk may be possible based on ncRNA expression levels.

Sex is also considered a major factor for AS, possibly due to hormone levels. Young women have a much lower risk of developing AS than men, and the risk of AS in postmenopausal women gradually increases or even exceeds that of men (Mathur et al., 2015). A study in a mouse AS model showed that miR-144 silencing prevented the development of AS in male mice but had no effect on female mice (Cheng et al., 2020). Thus, ncRNAs may play different roles in AS patients of different sexes, which is important to consider in the clinical treatment of AS. Strategies exist to improve lifestyle habits to prevent AS, and evidence suggests that ncRNA regulation is a key aspect of these strategies. Exercise is an effective measure to prevent AS, and exercise has been shown to downregulate lncRNA NEAT1 expression, protecting the endothelium from early AS (Yang Q. et al., 2023). In the aforementioned study, NEAT1 was found to induce apoptosis in endothelial cells by binding to KLF4, promoting the expression of the cellular focal death protein NLRP3. Exercise reduced the expression level of NEAT1 through N6-methyladenosine modification. There is a close relationship between diet and AS, and there is evidence that the intake of specific foods can reduce AS risk (Riccardi et al., 2022). Astaxanthin is a common nutrient that has been reported to have AS-protective effects, and a recent study demonstrated that the protective effect of astaxanthin on AS arises through the CircTPP2/miR-3073b-5p/ABCA1 axis that promotes macrophage cholesterol efflux and thus reduces foam cell formation (Zhang Z. et al., 2023). In addition, the side effects of some drugs used to treat other diseases may contribute to AS, and ncRNAs may be crucial regulators of these processes. The exploration of such mechanisms has the potential to improve the use of these drugs and identify new targets for treating AS. For example, after treatment with doxorubicin, a common chemotherapeutic agent with cardiac side effects that limit its application to some extent, miR-33 expression levels increased and consequently inhibited the expression level of ATP-binding cassette transporter protein A1 (ABCA1), promoting lipid accumulation in macrophages and exhibiting the hallmarks of early AS (Zhu et al., 2023). Another study demonstrated that inhibiting miR-33-5p can reduce plaque necrosis by regulating macrophage autophagy in AS, confirming the feasibility of targeting miR-33-5p in AS therapy (Ouimet et al., 2017). However, caution is needed on how to utilize miR-33 as a target for AS therapy, as silencing it for long periods of time may cause additional metabolic abnormalities (Goedeke et al., 2014). This is because miR-33 also plays an important role in cholesterol homeostasis (Marquart et al., 2010; Rayner et al., 2010). It is also noteworthy that two members of miR-33, miR-33a and miR-33b, showed different trends after statin treatment (Allen et al., 2012; Santovito et al., 2020b). Thus the formal application of miRNAs to the clinical treatment of AS has many challenges to overcome.

In summary, changes in ncRNA levels were observed in the presence of common risk factors for AS, further emphasizing the role of ncRNAs in AS development. Evidence suggests that aberrant ncRNA expression has substantial potential as a diagnostic basis for AS and that targeting aberrant ncRNAs to reverse such aberrant expression may have therapeutic effects on AS in the clinical setting.

AS is a non-fatal chronic disease that, without intervention, can easily worsen and lead to more serious CVDs. Abnormal ncRNA expression affects AS development. Many miRNAs, such as miR-217 and miR-124, regulate target mRNAs involved in AS development. As molecular sponges, lncRNAs and circRNAs can influence miRNA and mRNA expression through ceRNA in AS. These ncRNAs are potential biomarkers for the diagnostic screening and therapy of AS. Moreover, with the development of nanomolecular carriers, ncRNAs have become more effective as targeted therapies for AS.

## Abbreviations

ABCA1, ATP-binding cassette transporter protein A1; AS, atherosclerosis; CCL2, C-C motif chemokine ligand 2; CCR2, C-C chemokine receptor-2; ceRNA, competing endogenous RNA; CIA, common iliac artery; circRNA, circular RNA; CVD, Cardiovascular disease; DOX, doxorubicin; ICA, Icariside; LDL, low-density lipoprotein; lncRNA, long chain non coding RNA; MCP-1, monocyte chemotactic protein-1; miRNA, microRNA; MREs, miRNA responsive elements; ncRNA, non-coding RNA; PAM, peptide amphiphile micelle; pHLIP, pH low-insertion peptide; RISCs, RNA silencing complexes; UTMD, ultrasound-targeted microbubble destruction.
